# ﻿*Caloneisnanyiensis* sp. nov. (Bacillariophyta) from Nanyi Lake of Anhui Province, China

**DOI:** 10.3897/phytokeys.255.145664

**Published:** 2025-04-17

**Authors:** Yong Fang, Pan Yu, Qing-Min You, John Patrick Kociolek, Yue Cao, Quan-Xi Wang, Zhi-Ping Qian

**Affiliations:** 1 College of Life Sciences, Shanghai Normal University, Shanghai 200234, China Shanghai Normal University Shanghai China; 2 School of Environment and Geographical Sciences, Shanghai Normal University, Shanghai 200234, China Yangtze River Delta Urban Wetland Ecosystem National Field Scientific Observation and Research Station Shanghai China; 3 Yangtze River Delta Urban Wetland Ecosystem National Field Scientific Observation and Research Station, Shanghai 200234, China Shanghai Normal University shanghai China; 4 Museum of Natural History and Department of Ecology and Evolutionary Biology, University of Colorado, UCB 218, Boulder CO80309, USA University of Colorado Boulder United States of America; 5 Institute of Science and Technology, Shanghai Institute of Technology, Shanghai 201418, China Shanghai Institute of Technology Shanghai China

**Keywords:** Axial area, *Caloneis*, girdle, raphe, rhombic valve

## Abstract

A new species, *Caloneisnanyiensis***sp. nov.**, is described from Nanyi Lake, the largest lake in southern Anhui Province, China. Observations were made using a light and scanning electron microscope documenting the size, shape and ultrastructure of the new species. *Caloneisnanyiensis***sp. nov.** has rhombic valves with acutely rounded apices. The raphe is narrow and arched. An internal axial plate covers the alveoli, leaving small marginal openings bordered by costa, thickened and raised from the valve face. The striae are slightly radiate to parallel in the central area, becoming radiate towards the apices. By comparing the new species with similar *Caloneis* species, *C.nanyiensis***sp. nov.** was confirmed to be sufficiently different with respect to valve size and striae density to be recognised as new to science. The new species lives in freshwater habitats and epiphytic on *Cladophora*. The discovery enhances our knowledge of the diversity of freshwater diatoms in China.

## ﻿Introduction

Cleve and Grove initially described the genus *Caloneis* at the subgroup level, originally within the genus *Navicula* (Cleve and Grove 1891). Unfortunately, they did not provide a valid description; however, a few years later, Cleve provided a valid generic description in the *Synopsis of the Naviculoid Diatoms* (Cleve 1894). The type species of the genus *Caloneis* was indicated by Boyer (1927) to be *Caloneisamphisbaena* (Bory) Cleve (1894). The primary diagnostic features of this genus include valves that are linear-lanceolate to elliptical with capitate or rostrate ends. The shapes of axial and central areas are variable. Distal raphe fissures are usually distinct. The chambered striae give the appearance of one to two longitudinal lines. The striae of *Caloneis* are composed of fine alveoli. The outer wall of the alveoli is perforated by many rows of small, round poroids occluded by hymens and the inner wall of each alveolus typically opens to the inside of the valve by one fairly large, transapically elongate areola (Round et al. 1990). *Caloneis* is considered a widely distributed genus with a very broad ecological range, including freshwater, brackish and marine environments (Levkov and Williams 2014). To date, 392 taxonomically accepted species of *Caloneis* are listed in AlgaeBase (Guiry and Guiry 2025). After 2000, several new species have been reported from various localities from around the world (Lange-Bertalot et al. 2004; Metzeltin et al. 2005; Metzeltin and Lange-Bertalot 2007; Levkov and Williams 2014).

Traditionally, *Caloneis* and *Pinnularia* Ehrenberg have been considered distinct genera, the diagnostic features between the two genera mainly including the genus *Caloneis* having alveolate striae that are usually thinner and denser than those of *Pinnularia* (Hustedt 1935; Round et al. 1990; Mann 2001; Levkov and Williams 2014; Kulikovskiy et al. 2023). Molecular studies conducted thus far indicate that both the genus *Pinnularia* and the genus *Caloneis* are not monophyletic (Bruder et al. 2008; Souffreau et al. 2011; Kulikovskiy et al. 2023). These findings suggest that the genus *Pinnularia* may require further subdivision, which remains unresolved. With the in-depth study of molecular systematics, we believe that the taxonomic status of the genera *Pinnularia* and *Caloneis* will be more accurately and clearly divided.

In China, the vast majority of new *Caloneis* species were published before 2000, including Caloneisbacillumf.latilanceolatumZhu and Chen (1995), *Caloneischansiensis*Skvortzov (1935), C.elongatavar.constrictaCheng and Chin (1980), Caloneisfasciatavar.pekinensisSkvortzov (1928), Caloneisholstiivar.tibeticaJao (1964), *Caloneishunanensis* Chen and Zhu (Zhu and Chen 1989), Caloneislepidulavar.angustataSkvortzov (1976), Caloneispatagonicavar.sinicaSkvortzov (1938a), *C.platycephala*Cheng and Chin (1980), Caloneisschroderivar.densestriataSkvortzov (1976), Caloneisschumannianavar.biconstrictaf.minorZhu and Chen (1995), Caloneissiliculavar.hankensisSkvortzov (1929), Caloneisschumannianaf.gracilisSkvortzov (1935), Caloneissiliculavar.hinganicaSkvortzov (1976) and *Caloneissphagnicola*Skvortzov (1938b). After 2000, only one taxon has been reported in this genus: Caloneiscleveivar.parallela Skvortzov ex Gololobova and Kulikovskiy (Skvortzov 2012).

During the investigation of freshwater diatom diversity in Nanyi Lake, we discovered a new species, which is described here as *Caloneisnanyiensis* sp. nov. The purpose of this study was to document and formally describe the species, based on both light microscope (LM) and scanning electron microscope (SEM) observations and to compare it with other morphologically similar species of the genus.

## ﻿Material and methods

Diatom samples were collected from Nanyi Lake (31°01'–31°10'N, 118°50'–119°3'E), Anhui Province, China, in August 2018. The lake area is 210 km^2^. Nanyi Lake was formed by the differentiation of the ancient Danyang Lake. The ancient Danyang Lake system was a stagnant lake formed by long-term siltation and water accumulation in a newly-constructed fault depression. The climate of the Nanyi Lake area, as reported by the Langxi County meteorological station, belongs to the North subtropical monsoon humid climate zone. The main features are: mild climate, four distinct seasons, hot and rainy season, abundant rainfall and ample sunshine (Jia et al. 2021).

In the field, several water chemistry characteristics were recorded, including: pH, temperature, salinity, total dissolved solids (TDS) and conductivity. These parameters were measured using a YSIPro Plus multiparameter meter (YSI, Ohio, USA). In the field, collections of attached algae were scraped from the surfaces of stones using (sterilised?) toothbrushes and/or a knife and the samples were placed in a bottle, preserved with formalin (4% final concentration) and sealed.

In the laboratory, the diatom valves were cleaned of organic matter using the Microwave Accelerated Reaction System (Model MARS, CEM Corporation, USA) (Parr et al. 2004). The digestion followed a pre-programmed digestion scheme (temperature: 180 °C, ramp: 15 min, hold: 15 min) (Yu et al. 2019). After digestion, samples were alternatively centrifuged (5 minutes at 3500 rpm) and washed with distilled water (approximately five times) until the pH of the sample was approximately neutral. The cleaned material was kept in 95% ethanol. Cleaned diatom specimens were mounted on glass slides in Naphrax for light microscopy (LM) or air-dried on to coverslips and mounted on to Cu stubs for observation with a scanning electron microscope (SEM). LM studies were made with a ZEISS AXIO Imager A2 microscope fitted with DIC optics and a 1.4 numerical aperture, 100× oil immersion objective. SEM examination was conducted using a Hitachi SU-8010 (2 kV, working distance less than 6 mm). Images were compiled with Adobe Photoshop CS6. Morphological terminology follows Round et al. (1990). Both unprepared (field) samples and prepared slides of mounted material are housed in the Laboratory of Algae and Environment, Department of Biology, Shanghai Normal University (**SHTU**).

## ﻿Results

### ﻿ Caloneis nanyiensis

Taxon classificationPlantaeNaviculalesNaviculaceae

Pan Yu & Qing-Min You sp. nov.

3A75BA4F-2B8B-57F2-AA53-D574992C8856

Figs 1 , 2 , 3

#### Type material.

**Figure 1.**
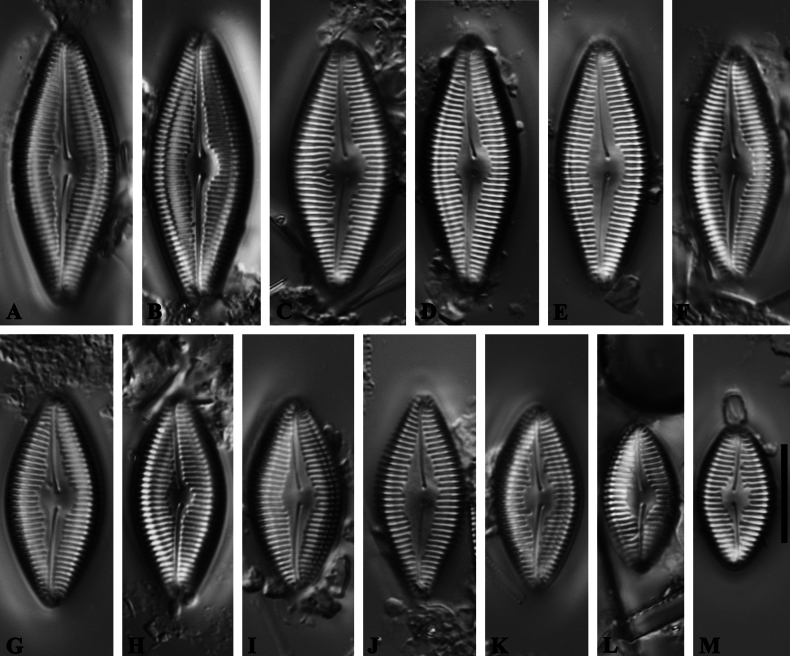
*Caloneisnanyiensis* sp. nov. LM ×1000 **A–M** thirteen valves showing a size diminution series; note that the valves rhombic and raphe narrow and arched. **A** Illustration of holotype specimen **D** illustration of isotype specimen. Scale bars: 10 µm (**M**).

***Holotype.*** Specimen circled on slide NYH-20180801 (= Fig. 1A), deposited in the Herbarium of Shanghai Normal University (SHTU), China.

***Isotype.*** Specimen circled on slide 652047 (= Fig. 1D), deposited in the Herbarium of University of Colorado, Boulder, USA.

#### Type locality.

China. Anhui Province: Nanyi Lake, sampling site with the coordinates 31°01'N, 118°50'E. Diatom samples collected by Pan Yu, 19 August 2018.

#### Description.

***LM*** (Fig. 1A–M): Valve length 14.5–28.5 μm, width 8.0–10.2 μm. Valves rhombical in shape with acutely rounded apices. Raphe narrow and arched, with slightly unilaterally bent central pores and slightly curved terminal fissures. Axial area linear-lanceolate, becoming wider at the central area. Striae are slightly radiate to parallel in the central area, becoming more radiate towards the apices, 16–18 in 10 μm.

**Figure 2.**
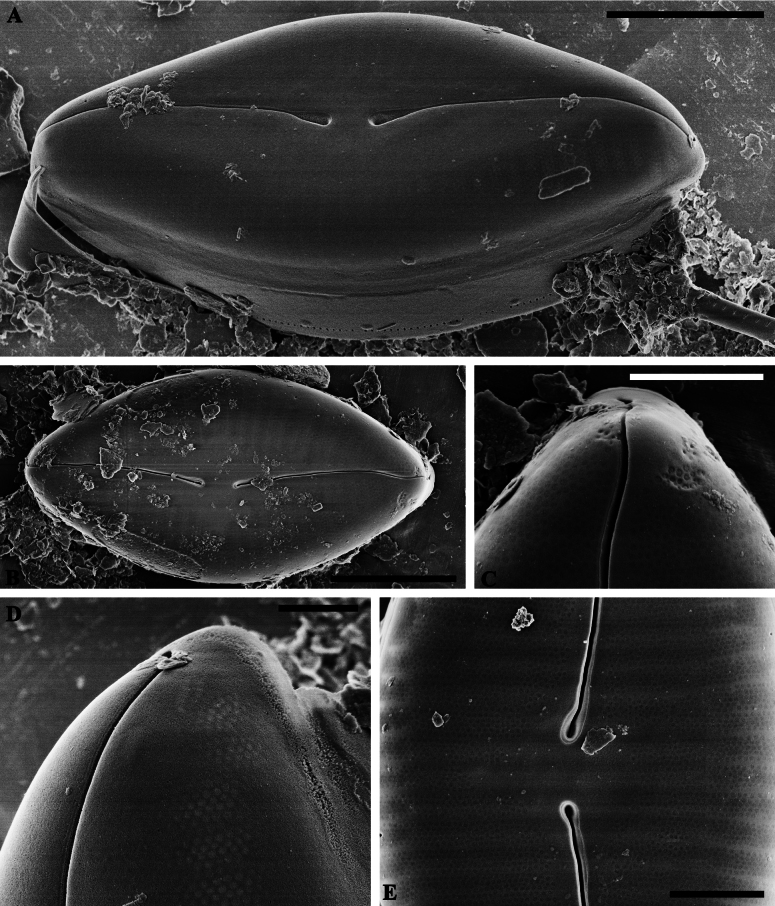
*Caloneisnanyiensis* sp. nov. SEM views of the external valve **A, B** external view of an entire valve note the raphe narrow and arched **C** details of the apices on the external valve; note the distal raphe fissures hooked and continuing on to the mantle **D** details of the striae on the external valve; note the Striae multiseriate, composed of small rounded areolae and areolae are occluded by heavy silicified, perforated hymenes **E** showing the central area. Scale bars: 5 µm (**A, B**); 2 µm (**C, E**); 1 µm (**D**).

***SEM valve exterior*** (Fig. 2A–E): Valve face usually uneven, with slightly raised ends and a slightly concave middle (Fig. 2A, B). Central area nearly rhombic, with a width accounting for 1/4–1/3 of the valve, the striae pattern in the central area is obviously shorter. (Fig. 2A, B, E). Raphe branches arched with proximal raphe ends weakly unilaterally deflected and dilated, drop-like in shape (Fig. 2A, B, E). Distal raphe fissures hooked and continuing on to the mantle (Fig. 2C, D). Striae multiseriate, composed of 4–5 rows of small, rounded areolae, which are occluded by heavy silicified, perforated hymenes (Fig. 2D, E). Girdle with a single row of linear poroids (Fig. 2A).

**Figure 3.**
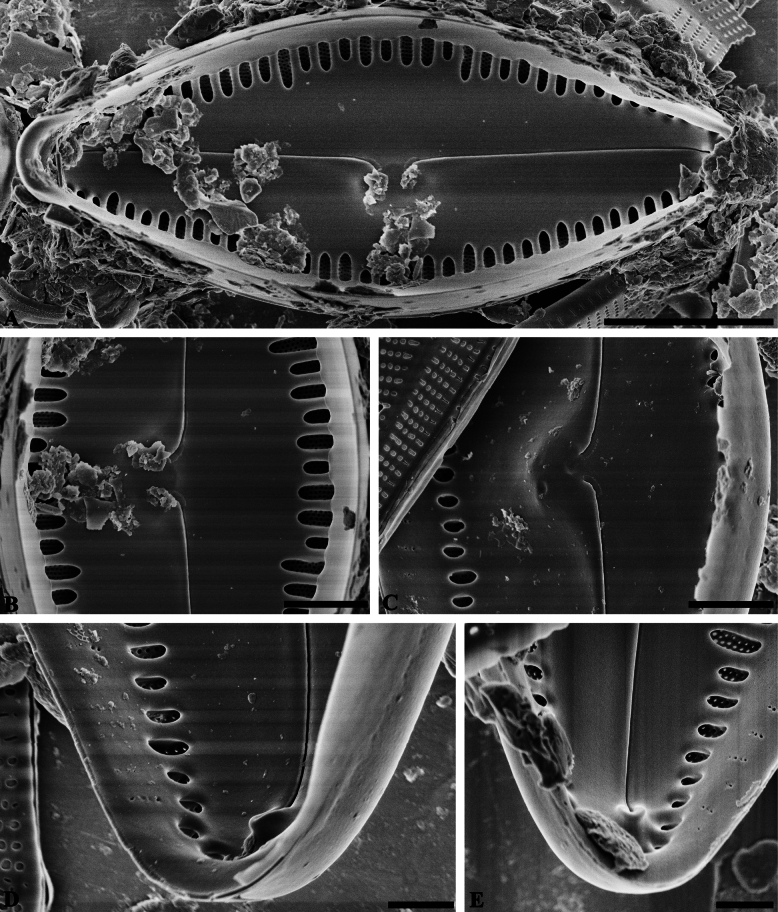
*Caloneisnanyiensis* sp. nov. SEM views of valve interior. **A** Internal view of an entire valve; note the raphe is filiform and slightly arched **B, C** details of the proximal raphe endings which are deflected to the same side **D, E** details of the distal raphe ends terminating as helictoglossae and the striae are composed of 2–4 areolae on the mantle. Scale bars: 5 µm (**A**); 2 µm (**B, C**); 1 µm (**D, E**).

***SEM valve interior*** (Fig. 3A–E): The raphe is filiform and slightly arched (Fig. 3A). The proximal raphe endings are deflected to the same sides (Fig. 3B, C) and curve towards a central nodule that is positioned to one side of the central area. The raphe terminates distally as an elevated helictoglossa (Fig. 3D, E). An axial plate covers the alveoli, leaving small marginal openings bordered by costae thickened and raised from the valve face (Fig. 3A, B). Striae composed of 2–4 areolae are present on the mantle (Fig. 3D, E).

#### Etymology.

The new species is named after the type locality, Nanyi Lake.

#### Distribution and ecology.

So far, the new species has only been collected at the type locality in Nanyi Lake. The habitat of the new species is characterised by pH 8.1, water temperature 30.2 °C, TDS 0.204 g.l^-1^, conductivity 175.7 μs.cm^-1^, collected in one sample (NYH–20180801) on *Cladophora*. In the type sample, this new species occurred at less than 2% relative abundance, established from a total count of 400 valves. Amongst the species co-occurring with *Caloneisnanyiensis* sp. nov., only four species accounted for more than 5% of the assemblage: *Fragilariapararumpens* Lange-Bertalot, Hofmann & Werum (Hofmann et al. 2013) (25.75%), *Achnanthidiumjackii* Rabenhorst (Rabenhorst 1861) (18.75%), *Aulacoseiragranulata* (Ehrenberg) Simonsen (Simonsen 1979) (11.5%) and *Encyonopsismicrocephala* (Grunow) Krammer (Krammer 1997) (%).

## ﻿Discussion

The new species described in this study possesses all the ultrastructural features of the genus *Caloneis* (Round et al. 1990), including having a narrow, slightly arched raphe with slightly unilaterally bent central pores and slightly curved terminal fissures, an axial plate covering the alveoli and striae parallel in the central area, becoming radiate towards the apices.

*Caloneisnanyiensis* sp. nov. can be compared to several species in the same genus, based on similarities in the outline and structure of the valve, including *C.caribeana* Metzeltin & Lange-Bertalot (Metzeltin and Lange-Bertalot 2007), *C.permagna* (Bailey) Cleve (Cleve 1894) and Caloneisschumannianavar.lancettula Hustedt (Hustedt 1930). The morphological characteristics of *C.nanyiensis* and these similar species are summarised in Table 1 to facilitate their comparison. The outline of the valves of *C.nanyiensis* is rhombical with acutely rounded ends, while that of *C.caribeana* is rhombical to rhombic-lanceolate with acutely rounded ends, *C.permagna* is rhombic-lanceolate with acutely rounded ends and C.schumannianavar.lancettula is narrow-lanceolate with broadly rounded ends. Additionally, the valves of *C.nanyiensis* are shorter (14.5–28.5 μm) than *C.permagna* (85–220 μm), *C.caribeana* (32–57 μm) and C.schumannianavar.lancettula (35–40 μm), as well as narrower (8.0–10.2 μm in *C.nanyiensis*) versus the breadth of valves in *C.permagna* (35–55 μm) and *C.caribeana* (14–16 μm). Furthermore, no central area was observed in the new species, but *C.caribeana* has a narrow fascia central area, *C.permagna* has an irregularly lanceolate central area and C.schumannianavar.lancettula has a transapically rectangular central area. The stria density of *C.nanyiensis* is higher (16–18/10 μm) than that of *C.permagna* (9–12/10 μm), while being lower than those of the other similar species. Individuals in the populations of *C.nanyiensis* examined here have a valve outline that is rhombical in shape, which, together with the slightly curved raphe, help to differentiate it from other species in the genus.

**Table 1.** Comparison of morphological characteristics of *Caloneisnanyiensis* sp. nov. and closely-related taxa.

Species/Feature	*C.nanyiensis* sp. nov.	*C.caribeana* Metzeltin & Lange-Bertalot	*C.permagna* (Bailey) Cleve	C.schumannianavar.lancettula Hustedt
Valve length (μm)	14.5–28.5	32–57	85–220	35–40
Valve width (μm)	8.0–10.2	14–16	35–55	8
Valve outline	Rhombical	Rhombical to rhombic-lanceolate	Rhombical-lanceolate	Narrow-lanceolate
Valve apices	Acutely round	Acutely round	Acutely round	Broadly rounded
Axial area	Linear to lanceolate	Narrow, linear	Irregularly lanceolate	Narrow-lanceolate
Central area	Absent	Very narrow fascia	Irregularly lanceolate	Transapically rectangular
Raphe	Narrow and arched	Distinctly curved	Straight	Straight
Density of striae (10 μm)	16–18	17–20	9–12	17–20
References	Current study	Metzeltin and Lange-Bertalot (2007)	Cleve (1894), Krammer and Lange-Bertalot (1986)	Hustedt (1930), Krammer and Lange-Bertalot (1986)

We also compared our new species with the smaller valves of *C.distinguenda* Levkov & Williams (Levkov and Williams 2014). However, the latter taxon is characterised by elliptic-lanceolate valves being 36 μm long and 15 μm wide. Additionally, the central area in smaller specimens of *C.distinguenda* is distinctly separated from axial area and transversally elliptic in shape. The density of the striae in smaller specimens of *C.distinguenda* is lower (14–16/10 μm) than that of *C.nanyiensis*.

*Caloneis* are common in alkaline, brackish and marine habitats, species of the genus *Caloneis* having a broad ecological niche (Levkov and Williams 2014). For example, *Caloneis* is diverse in cold-water oligotrophic habitats, *C.limosa* prefers to inhabit alkaliphilic, oligotrophic and up to β-mesosaprobic water bodies, and *C.schumanniana* is classified as a planktonic-benthic, oligo-xenosaprobiontic and alkaliphilic species (Denys 1991; Stancheva et al. 2009; Levkov and Williams 2014). In the present study, *Caloneisnanyiensis* has only been found on *Cladophora* in Nanyi Lake. The water quality of Nanyi Lake reflects a moderate nutrient level. This investigation did not detect the presence of this novel species in any other location. It is postulated that this species may be a good indicator of water bodies with moderate nutrient levels. However, further ecological studies are needed to confirm this hypothesis.

## Supplementary Material

XML Treatment for Caloneis nanyiensis

## ﻿Additional information

### Author ORCIDs

Pan Yu https://orcid.org/0000-0001-7937-2381

John Patrick Kociolek https://orcid.org/0000-0001-9824-7164

Zhi-Ping Qian https://orcid.org/0009-0008-7514-8073

### Data availability

All of the data that support the findings of this study are available in the main text.
